# 1,3-Bis(phenyl­sufanylmeth­yl)benzene

**DOI:** 10.1107/S1600536810005775

**Published:** 2010-02-20

**Authors:** Constantine A. Stewart, Diane A. Dickie, Richard A. Kemp

**Affiliations:** a Advanced Materials Laboratory, Sandia National Laboratories, 1001 University Blvd SE, Albuquerque, NM, 87106, USA; b Department of Chemistry and Chemical Biology, MSC03 2060, 1 University of New Mexico, Albuquerque, NM, 87131, USA

## Abstract

The complete mol­ecule of the title compound, C_20_H_18_S_2_, is generated by crystallographic mirror symmetry, with two C atoms lying on the mirror plane. All of the independent atoms are contained within two planes defined by the thio­phenyl rings (C_6_S) and the central phenyl ring with the methyl­ene bridge; the r.m.s deviations of these planes are 0.012 and 0.025 Å, respectively. The two planes are almost perpendicular to one another at a dihedral angle of 80.24 (10)°. Inter­molecular C—H—π inter­actions are present in the crystal structure.

## Related literature

For the use of the title compound as a ligand, see: Bu *et al.* (2002 ▶); Romero *et al.* (1996 ▶); Loeb & Wisner (1998 ▶); Kruithof *et al.* (2008 ▶); Bergholdt *et al.* (1998 ▶); Kobayashi *et al.* (2000 ▶). For related organic mol­ecules, see: Cervantes *et al.* (2006 ▶); Sillanpää *et al.* (1994 ▶); Arroyo *et al.* (2003 ▶).
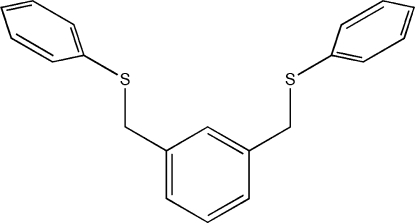


## Experimental

### 

#### Crystal data

C_20_H_18_S_2_
*M* *_r_* = 322.46
Orthorhombic, 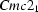

*a* = 32.072 (2) Å
*b* = 7.6053 (5) Å
*c* = 6.8224 (5) Å
*V* = 1664.1 (2) Å^3^
*Z* = 4
Mo *K*α radiation
μ = 0.31 mm^−1^
*T* = 228 K
0.76 × 0.15 × 0.10 mm

#### Data collection

Bruker APEXII CCD area-detector diffractometer
Absorption correction: multi-scan (*SADABS*; Bruker, 2001 ▶) *T*_min_ = 0.797, *T*_max_ = 0.970
14404 measured reflections
1689 independent reflections
1417 reflections with *I* > 2σ(*I*)
*R*_int_ = 0.052

#### Refinement

*R*[*F* ^2^ > 2σ(*F* ^2^)] = 0.035
*wR*(*F* ^2^) = 0.086
*S* = 1.12
1689 reflections
103 parameters
1 restraint
H-atom parameters constrained
Δρ_max_ = 0.27 e Å^−3^
Δρ_min_ = −0.31 e Å^−3^
Absolute structure: Flack (1983 ▶), 738 Friedel pairs
Flack parameter: 0.05 (12)

### 

Data collection: *APEX2* (Bruker, 2007 ▶); cell refinement: *SAINT* (Bruker, 2007 ▶); data reduction: *SAINT*; program(s) used to solve structure: *XS* in *SHELXTL* (Sheldrick, 2008 ▶); program(s) used to refine structure: *XL* in *SHELXTL*; molecular graphics: *DIAMOND* (Brandenburg, 2007 ▶); software used to prepare material for publication: *publCIF* (McMahon & Westrip, 2008 ▶) and *PLATON* (Spek, 2009 ▶).

## Supplementary Material

Crystal structure: contains datablocks I, New_Global_Publ_Block. DOI: 10.1107/S1600536810005775/fb2176sup1.cif

Structure factors: contains datablocks I. DOI: 10.1107/S1600536810005775/fb2176Isup2.hkl

Additional supplementary materials:  crystallographic information; 3D view; checkCIF report

## Figures and Tables

**Table 1:** Hydrogen-bond geometry (Å, °) *Cg*1 and *Cg*2 are the centroids of the C1–C4/C3′/C2′ and C6–C11 rings, respectively.

*D*—H⋯*A*	*D*—H	H⋯*A*	*D*⋯*A*	*D*—H⋯*A*
C1—H1*A*⋯*Cg*1^i^	0.94	2.69	3.57 (5)	155
C1—H1*A*⋯*Cg*1^ii^	0.94	2.69	3.57 (5)	155
C7—H7*A*⋯*Cg*2^iii^	0.94	2.85	3.67 (6)	147

Symmetry codes: (i) 
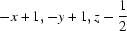
; (ii) 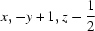; (iii) 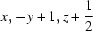.

**Fig. 1.**
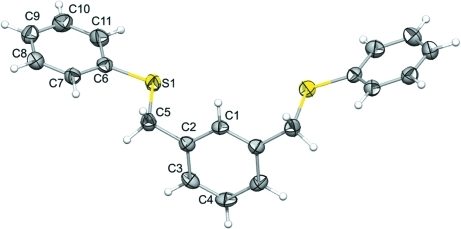
View of the title compound showing full numbering scheme. The displacement ellipsoids are shown at the 50% probability level.

## supplementary crystallographic information

### Comment

The title compound, 1,3-bis(phenylthiomethyl)benzene, belongs to a class of transition metal ligands known as SCS-pincer ligands because of their tendency to act as tridentate chelators through one carbon and two sulfur atoms. The plane containing the thiophenyl ring is rotated almost perpendicular to the plane of the central phenyl ring, at an angle of 80.24 (10)°. This is comparable to the para-fluoro (Cervantes *et al.*, 2006) and ortho-ethylester (Sillanpää *et al.*, 1994) derivatives, whose interplanar angles measure 78.13 (10) and 87.66 (10)° respectively. Surprisingly, in the only other structurally characterized derivative, the perfluorinated 1,3-bis(pentafluorophenylthiomethyl)benzene (Arroyo *et al.*, 2003), the rings are nearly co-planar with angles of 8.93 (10) and 10.85 (9)° between the central phenyl and the two independent flanking perfluoro rings. In both the title compound and the para-fluoro derivative, the flanking aryl rings are oriented on the same face of the central ring.

Although the structure of the title compound has not been previously determined, several of its metal complexes are known. Two types of coordination were observered in structurally characterized metal complexes. In the presence of silver perchlorate (Bu *et al.*, 2002) or silver nitrate (Romero *et al.*, 1996), the title compound acts as a bridging ligand through sulfur. In these two complexes, the flanking aryls remain on the same face of the central ring. With Pd (Loeb & Wisner, 1998), Pt (Kruithof *et al.*, 2008) and Te (Bergholdt *et al.*, 1998; Kobayashi *et al.*, 2000) the much more common trihapto-SCS "pincer"-type coordination is observed and the thiophenyl rings rotate to opposite faces of the central phenyl. In all seven examples, the mirror plane present within the free ligand has been broken. The nearly perpendicular orientation of the planes of flanking aryls and the central phenyl (80.24 (10)°) is maintained in each of the Te (Bergholdt *et al.*, 1998; Kobayashi *et al.*, 2000) and Pt (Kruithof *et al.*, 2008) complexes, which have interplanar angles ranging from 77.8 (3) to 89.7 (3)°. In contrast, the interplanar angles in the Ag and Pd complexes cover a much wider range within and across the molecules, measuring 44.1 (6) and 65.5 (6)° in the AgClO_4_ based-complex (Bu *et al.*, 2002), 44.5 (3) and 70.4 (2)° for AgNO_3_ (Romero *et al.*, 1996), and 40.3 (12) and 88.8 (13)° at one end of the Pd-rotaxane and 41.9 (12) and 89.4 (13)° at the other (Loeb & Wisner, 1998).

### Experimental

1,3-Bis((phenylthio)methyl)benzene was synthesized according to the literature method (Romero, *et al.* 1996). To grow crystals, a saturated solution of 1,3-bis((phenylthio)methyl)benzene was prepared in hot pentane. An aliquot of this saturated solution was transferred to a 20 ml vial, which was then tightly capped. The vial was placed in a bath of hot water and allowed to slowly cool to room temperature. Colorless, needle-like crystals of up to 10 mm in length formed over approximately 30 minutes. The solvent was then decanted and the crystals allowed to air dry. Several needles of ca. 1 mm in length were placed in a melting point capillary with an internal diameter 0.5 mm and the melting point was determined to be 84 - 86 °C.

### Refinement

Hydrogen atoms were included at geometrically idealized positions with C—H distances of 0.94 Å for aryl H atoms and 0.98 Å for methylene H atoms. The hydrogen atoms were treated as riding on their respective heavy atoms. The isotropic thermal parameters of the hydrogen atoms were fixed at 1.2 *U*_eq_ of the parent atom.

### Crystal data

**Table:**

C_20_H_18_S_2_	*D*_x_ = 1.287 Mg m^−^^3^
*M**_r_* = 322.46	Melting point = 357–359 K
Orthorhombic, *C**m**c*2_1_	Mo *K*α radiation, λ = 0.71073 Å
Hall symbol: C 2c -2	Cell parameters from 8194 reflections
*a* = 32.072 (2) Å	θ = 2.8–28.1°
*b* = 7.6053 (5) Å	µ = 0.31 mm^−^^1^
*c* = 6.8224 (5) Å	*T* = 228 K
*V* = 1664.1 (2) Å^3^	Needle, colourless
*Z* = 4	0.76 × 0.15 × 0.10 mm
*F*(000) = 680	

### Data collection

**Table:**

Bruker APEXII CCD area-detector diffractometer	1689 independent reflections
Radiation source: fine-focus sealed tube	1417 reflections with *I* > 2σ(*I*)
graphite	*R*_int_ = 0.052
φ and ω scans	θ_max_ = 26.4°, θ_min_ = 3.3°
Absorption correction: multi-scan (*SADABS*; Bruker, 2001)	*h* = −40→40
*T*_min_ = 0.797, *T*_max_ = 0.970	*k* = −9→9
14404 measured reflections	*l* = −8→8

### Refinement

**Table:**

Refinement on *F*^2^	Secondary atom site location: difference Fourier map
Least-squares matrix: full	Hydrogen site location: difference Fourier map
*R*[*F*^2^ > 2σ(*F*^2^)] = 0.035	H-atom parameters constrained
*wR*(*F*^2^) = 0.086	*w* = 1/[σ^2^(*F*_o_^2^) + (0.0408*P*)^2^ + 0.6358*P*] where *P* = (*F*_o_^2^ + 2*F*_c_^2^)/3
*S* = 1.12	(Δ/σ)_max_ < 0.001
1689 reflections	Δρ_max_ = 0.27 e Å^−^^3^
103 parameters	Δρ_min_ = −0.31 e Å^−^^3^
1 restraint	Absolute structure: Flack (1983), 736 Friedel pairs
38 constraints	Flack parameter: 0.05 (12)
Primary atom site location: structure-invariant direct methods	

### Special details

**Table:**

Geometry. All esds (except the esd in the dihedral angle between two l.s. planes) are estimated using the full covariance matrix. The cell esds are taken into account individually in the estimation of esds in distances, angles and torsion angles; correlations between esds in cell parameters are only used when they are defined by crystal symmetry. An approximate (isotropic) treatment of cell esds is used for estimating esds involving l.s. planes.
Refinement. Refinement of *F*^2^ against ALL reflections. The weighted *R*-factor wR and goodness of fit *S* are based on *F*^2^, conventional *R*-factors *R* are based on *F*, with *F* set to zero for negative *F*^2^. The threshold expression of *F*^2^ > σ(*F*^2^) is used only for calculating *R*-factors(gt) etc. and is not relevant to the choice of reflections for refinement. *R*-factors based on *F*^2^ are statistically about twice as large as those based on *F*, and *R*- factors based on ALL data will be even larger.

### Fractional atomic coordinates and isotropic or equivalent isotropic displacement parameters (Å^2^)

**Table:**

	*x*	*y*	*z*	*U*_iso_*/*U*_eq_	
S1	0.415866 (15)	0.21909 (7)	0.49099 (13)	0.03480 (17)	
C1	0.5000	0.3639 (4)	0.7139 (5)	0.0295 (7)	
H1A	0.5000	0.4312	0.5984	0.035*	
C2	0.46220 (7)	0.3148 (3)	0.7983 (4)	0.0295 (5)	
C3	0.46271 (7)	0.2166 (3)	0.9699 (5)	0.0362 (6)	
H3A	0.4374	0.1836	1.0290	0.043*	
C4	0.5000	0.1671 (4)	1.0546 (6)	0.0406 (9)	
H4A	0.5000	0.0996	1.1700	0.049*	
C5	0.42152 (7)	0.3621 (3)	0.7015 (4)	0.0343 (6)	
H5A	0.4218	0.4855	0.6601	0.041*	
H5B	0.3983	0.3448	0.7928	0.041*	
C6	0.36379 (7)	0.2480 (3)	0.4147 (4)	0.0313 (5)	
C7	0.33665 (6)	0.3748 (3)	0.4885 (5)	0.0344 (5)	
H7A	0.3459	0.4547	0.5843	0.041*	
C8	0.29608 (8)	0.3827 (3)	0.4201 (4)	0.0400 (6)	
H8A	0.2779	0.4681	0.4710	0.048*	
C9	0.28185 (8)	0.2686 (3)	0.2796 (4)	0.0449 (7)	
H9A	0.2541	0.2750	0.2353	0.054*	
C10	0.30905 (8)	0.1432 (4)	0.2032 (4)	0.0467 (7)	
H10A	0.2998	0.0651	0.1058	0.056*	
C11	0.34957 (8)	0.1333 (3)	0.2704 (4)	0.0401 (6)	
H11A	0.3677	0.0483	0.2183	0.048*	

### Atomic displacement parameters (Å^2^)

**Table:**

	*U*^11^	*U*^22^	*U*^33^	*U*^12^	*U*^13^	*U*^23^
S1	0.0321 (3)	0.0375 (3)	0.0348 (3)	0.0052 (2)	0.0004 (3)	−0.0071 (4)
C1	0.0353 (17)	0.0270 (15)	0.0264 (19)	0.000	0.000	0.0012 (14)
C2	0.0326 (12)	0.0268 (10)	0.0290 (13)	0.0000 (9)	0.0007 (10)	−0.0049 (11)
C3	0.0406 (12)	0.0326 (10)	0.0356 (15)	−0.0048 (9)	0.0095 (14)	−0.0008 (13)
C4	0.058 (2)	0.0348 (17)	0.029 (2)	0.000	0.000	0.0085 (15)
C5	0.0304 (11)	0.0341 (12)	0.0382 (15)	0.0018 (10)	0.0014 (11)	−0.0071 (11)
C6	0.0316 (12)	0.0328 (11)	0.0295 (13)	−0.0041 (9)	0.0021 (9)	0.0027 (10)
C7	0.0321 (10)	0.0384 (11)	0.0327 (13)	−0.0010 (8)	0.0007 (14)	−0.0038 (15)
C8	0.0355 (13)	0.0476 (14)	0.0369 (15)	0.0035 (11)	0.0043 (11)	−0.0001 (11)
C9	0.0335 (13)	0.0581 (15)	0.0430 (18)	−0.0060 (11)	−0.0051 (12)	0.0049 (15)
C10	0.0478 (16)	0.0509 (16)	0.0413 (18)	−0.0095 (12)	−0.0065 (13)	−0.0095 (13)
C11	0.0446 (14)	0.0375 (13)	0.0381 (16)	0.0002 (11)	0.0010 (12)	−0.0101 (12)

### Geometric parameters (Å, °)

**Table:**

S1—C6	1.763 (2)	C5—H5B	0.9800
S1—C5	1.811 (2)	C6—C11	1.392 (3)
C1—C2^i^	1.393 (3)	C6—C7	1.393 (3)
C1—C2	1.393 (3)	C7—C8	1.384 (3)
C1—H1A	0.9400	C7—H7A	0.9400
C2—C3	1.388 (4)	C8—C9	1.371 (4)
C2—C5	1.506 (3)	C8—H8A	0.9400
C3—C4	1.381 (3)	C9—C10	1.394 (4)
C3—H3A	0.9400	C9—H9A	0.9400
C4—C3^i^	1.381 (3)	C10—C11	1.380 (3)
C4—H4A	0.9400	C10—H10A	0.9400
C5—H5A	0.9800	C11—H11A	0.9400
			
C6—S1—C5	104.72 (11)	C11—C6—C7	119.0 (2)
C2^i^—C1—C2	121.0 (3)	C11—C6—S1	116.16 (18)
C2^i^—C1—H1A	119.5	C7—C6—S1	124.85 (19)
C2—C1—H1A	119.5	C8—C7—C6	119.7 (2)
C3—C2—C1	118.8 (2)	C8—C7—H7A	120.2
C3—C2—C5	120.57 (19)	C6—C7—H7A	120.2
C1—C2—C5	120.5 (2)	C9—C8—C7	121.4 (2)
C4—C3—C2	120.6 (2)	C9—C8—H8A	119.3
C4—C3—H3A	119.7	C7—C8—H8A	119.3
C2—C3—H3A	119.7	C8—C9—C10	119.1 (2)
C3—C4—C3^i^	120.1 (4)	C8—C9—H9A	120.5
C3—C4—H4A	120.0	C10—C9—H9A	120.5
C3^i^—C4—H4A	120.0	C11—C10—C9	120.2 (2)
C2—C5—S1	106.92 (15)	C11—C10—H10A	119.9
C2—C5—H5A	110.3	C9—C10—H10A	119.9
S1—C5—H5A	110.3	C10—C11—C6	120.6 (2)
C2—C5—H5B	110.3	C10—C11—H11A	119.7
S1—C5—H5B	110.3	C6—C11—H11A	119.7
H5A—C5—H5B	108.6		
			
C2^i^—C1—C2—C3	0.6 (4)	C5—S1—C6—C7	8.5 (2)
C2^i^—C1—C2—C5	−177.45 (19)	C11—C6—C7—C8	1.1 (4)
C1—C2—C3—C4	−0.6 (4)	S1—C6—C7—C8	−178.7 (2)
C5—C2—C3—C4	177.4 (2)	C6—C7—C8—C9	−0.4 (4)
C2—C3—C4—C3^i^	0.7 (5)	C7—C8—C9—C10	−0.5 (4)
C3—C2—C5—S1	−104.0 (2)	C8—C9—C10—C11	0.7 (4)
C1—C2—C5—S1	74.0 (2)	C9—C10—C11—C6	0.0 (4)
C6—S1—C5—C2	168.24 (15)	C7—C6—C11—C10	−0.9 (4)
C5—S1—C6—C11	−171.28 (19)	S1—C6—C11—C10	178.9 (2)

Symmetry codes: (i) −*x*+1, *y*, *z*.

### Hydrogen-bond geometry (Å, °)

**Table:**

Cg1 and Cg2 are the centroids of the C1–C4/C3'/C2' and C6–C11 rings, respectively.

**Table:**

*D*—H···*A*	*D*—H	H···*A*	*D*···*A*	*D*—H···*A*
C1—H1A···Cg1^ii^	0.94	2.69	3.57 (5)	155
C1—H1A···Cg1^iii^	0.94	2.69	3.57 (5)	155
C7—H7A···Cg2^iv^	0.94	2.85	3.67 (6)	147

Symmetry codes: (ii) −*x*+1, −*y*+1, *z*−1/2; (iii) *x*, −*y*+1, *z*−1/2; (iv) *x*, −*y*+1, *z*+1/2.
